# Evaluating Treatment Outcomes in Women with Node-Negative T1 Breast Cancers

**DOI:** 10.3390/cancers16244228

**Published:** 2024-12-19

**Authors:** Patrick Mun Yew Chan, Kay Hsiang Ong, Sherwin Kuah, E Jan Sim, Juliana Chen, Mui Heng Goh, Wei-Wen Ang, Ern Yu Tan

**Affiliations:** 1 Department of General Surgery, Tan Tock Seng Hospital, Singapore 308433, Singapore; patrick_chan@ttsh.com.sg (P.M.Y.C.); sherwin_kuah@ttsh.com.sg (S.K.); ejan_sim@ttsh.com.sg (E.J.S.); juliana_chen@ttsh.com.sg (J.C.); mui_heng_goh@ttsh.com.sg (M.H.G.); wei-wen_ang@ttsh.com.sg (W.-W.A.); 2 Lee Kong Chian School of Medicine, Singapore 308232, Singapore; kayhsiang.ong@mohh.com.sg; 3 Institute of Molecular and Cell Biology, A*STAR, Singapore 138673, Singapore

**Keywords:** node negative breast cancer, chemotherapy, survival

## Abstract

Background: With greater awareness and increased screening, cancers are increasingly being diagnosed at stage I. Women with these small node-negative tumours have excellent survival prospects after surgery, but many women, especially those with triple-negative and human epidermal growth factor receptor (HER)-2-positive tumours, still receive adjuvant systemic treatments to reduce the recurrence risk. Aims: We review the outcomes of women diagnosed with stage I (T1N0M0) tumours in our unit and examine the effect of systemic chemotherapy with/without targeted therapy on recurrence patterns and survival outcomes. Results: We reviewed 643 women diagnosed with T1N0M0 disease over a 10-year period. Five-year recurrence-free survival (RFS) was 96.6% and the 10-year RFS was 95.5%. Recurrence occurred in 4.7% of the women and was limited to locoregional sites in two-thirds of the instances. Systemic recurrences developed in 12 women, all of whom had ER-positive/HER2-negative disease. The mode of surgery emerged as the only independent predictor of recurrence. Recurrence was highest in women treated with wide local excision (WLE) alone (*p* < 0.05), but not in those who had received breast radiation after WLE (*p* = 0.112). Systemic chemotherapy, with or without anti-HER2 therapy, was discussed with 334 women, of whom 50.6% received the treatment; these women were more often younger and had triple-negative or HER2-positive tumours (*p* < 0.001). Women who received chemotherapy showed a non-significant tendency to develop locoregional recurrence (*p* = 0.104), but the number of systemic recurrences were similar to those documented in women who had not received chemotherapy. Chemotherapy and/or targeted treatment was not observed to have a significant effect on 5-year recurrence-free survival (*p* = 0.444). Conclusions: Stage I cancers have excellent survival outcomes. An optimal local surgical treatment is important and we did not find chemotherapy and/or targeted therapy to produce any significant differences in survival.

## Simple Summary

This manuscript reports on the treatment outcomes associated with T1N0 breast cancers, the incidence of which is rising as more women attend screening and seek medical attention early. Some women fare poorly, but most have a good prognosis after surgery and recurrence event rates are low. This makes it particularly important to optimise systemic treatment recommendations so as to adequately treat women with poor-risk tumours, sparing the majority from over-treatment.

## 1. Introduction

More cancers are being diagnosed in the early stages because of greater public awareness and more widespread acceptance of breast cancer screening. The increased sensitivity of newer breast imaging modalities has also contributed to more cancers being detected when they are smaller. Over the past decade, almost a third of breast cancers have been diagnosed at stage I, defined as tumours smaller than 2 cm in size (T1) and without nodal involvement (N0) [1,2]. Prognosis is excellent after surgery, especially with T1a and T1b tumours [3], and breast-cancer-specific survival rates are frequently reported in the region of 95% and 90% after 5 years and 10 years, respectively [4,5]. However, a handful of women will have a relapse and it is for this reason that adjuvant treatments are considered. Recurrences limited to locoregional sites are generally amenable to curative treatment and the prognosis remains good. This contrasts with systemic recurrence, for which treatment is often palliative. Chemotherapy and targeted therapy are directed against micro-metastases, which are likely more prevalent in human epidermal growth factor receptor (HER)2-positive and triple-negative tumours, given the higher rates of systemic recurrence in tumours of these subtypes [6,7]. Current NCCN guidelines recommend adjuvant chemotherapy and targeted therapy for T1c human epidermal growth factor receptor (HER)2-positive and triple-negative tumours, and, increasingly, more women with T1b tumours are also receiving this treatment. Even in the context of T1a diseases, systemic chemotherapy and targeted therapy are now being discussed.

Recommendations for adjuvant chemotherapy in oestrogen receptor (ER)-positive/HER2-positive tumours were previously no different. Chemotherapy used to be offered to women with T1c tumours and discussed with those with T1b tumours, but this has changed since the advent of genomic assay testing as the standard of care in many tertiary breast units. Regardless of the specific assay used, the data consistently show that a considerable proportion of women with node-negative ER-positive/HER2-negative tumours have a low risk of recurrence and derive little benefit from the addition of chemotherapy to the standard hormonal therapy [8]. This has spared many women from unnecessary treatment and illustrates how tumour profiling has facilitated the identification of high-risk subgroups for more tailored treatments. However, there is still no reliable means of risk stratifying HER2-positive and triple-negative tumours and, consequently, there is a push towards chemotherapy and targeted therapy even for the treatment of small tumours, largely because of the proven efficacy of anti-HER2 therapies in the former and the lack of any other systemic alternative in the latter.

While chemotherapy treatments have been de-escalated in relation to hormone-positive/HER2-negative tumours, the opposite seems to be the case with HER2-positive and triple-negative tumours, where treatment is now discussed even with women with T1a disease. Prospective and randomised studies are challenging to perform in relation to T1 tumours, as the low frequency of events mean that large sample sizes and a long follow-up period are required to achieve sufficient statistical power. Recruitment into such studies can be difficult as well, as many women with the T1N0 disease consider themselves ‘cured’ after surgery and fail to understand the rationale for systemic chemotherapy. In this study, we have reviewed women diagnosed with stage I (T1N0M0) tumours at our unit to examine the recurrence patterns and evaluate the impact of adjuvant systemic chemotherapy and anti-HER2 therapy on survival outcomes. We also reviewed the prevailing practices for chemotherapy and anti-HER2 therapy recommendations at our unit and sought to characterise the women who agreed to these treatments. Our study cohort comes from a period when genomic testing was seldom performed for ER-positive/HER2-negative tumours, when dual anti-HER2 blockade was not used, and when 5 years of hormonal therapy was the standard of care.

## 2. Methods

A retrospective study of women with T1N0M0 breast cancer diagnosed between 2006 and 2016 at a single breast unit in a tertiary public hospital in Singapore was conducted. Institutional review board approval was obtained for this study (DSRB 2019/00217).

Inclusion criteria. Patients diagnosed with stage I or T1N0M0 tumours, as per the seventh edition of the American Joint Committee on Cancer (AJCC) staging system 2010, were identified from our prospective database. Only tumours with no nodal involvement (either on sentinel lymph node biopsy or axillary clearance) were included.

Exclusion criteria. Patients who were staged as T1N0 after having received a neoadjuvant treatment and patients with systemic metastases found on staging scans. No patient had received any neoadjuvant treatment.

Tumour classification. Tumours reported to have a maximum diameter of 20 mm or smaller following histopathological analyses were defined as T1; further classified as T1a (more than 1 mm and up to 5 mm), T1b (6 mm to 10 mm), and T1c (11 mm to 20 mm). Tumours staged as T1mic (microinvasive foci of less than 1 mm) and Tis (in situ carcinoma, with no foci of invasion) were excluded. Only tumours with no nodal involvement (either on sentinel lymph node biopsy or axillary clearance) were included, and staging scans were not performed in these cases unless there were specific concerns. Tumours were classified according to their receptor status: (i) triple-negative tumours lacking ER, progesterone receptor (PR) and HER2 expression, (ii) HER2-positive, regardless of ER or PR status, and (iii) ER-positive/HER2-negative. Both ER and PR were considered negative if less than 1% of the cells stained positive. The HER2 status was considered to be negative if the immunohistochemistry score was 0 to 1+ and if there was no amplification of fluorescence in situ hybridization (FISH) relative to cases with a 2+ immunohistochemical score.

Surgery and adjuvant treatment recommendations. All women underwent surgery as the first treatment. The decision to perform a mastectomy or WLE was made based on medical suitability and according to patient preferences. Following surgery, all cases were discussed at the weekly multidisciplinary tumour board meetings, where recommendations for adjuvant treatments, including chemotherapy, anti-HER2 therapy, hormonal therapy, and radiation, were made. Whole breast radiation with a boost to the tumour bed was standard for all women who received WLE. Radiation was not indicated if a mastectomy had been done. Systemic chemotherapy and targeted anti-HER2 therapy recommendations were made in accordance with the prevailing NCCN guidelines: recommended for T1c tumours, discussed in relation to T1b tumours, and not indicated for T1a tumours, unless there were numerous risk factors that increased the risk of recurrence. Chemotherapy regimens were used in accordance with oncologist preferences and included both anthracycline-based (anthracycline/cyclophosphamide and paclitaxel) and non-anthracycline (docetaxel/cyclophosphamide) regimens. Dose-dense regimens were not used. Single-agent trastuzumab was the anti-HER2 therapy in use and was administered in combination with paclitaxel. Hormonal therapy was generally recommended for a duration of 5 years; the aromatase inhibitor (AI) was the preferred agent for use in post-menopausal women, while tamoxifen was the preferred agent for use in pre-menopausal women. The final decision for adjuvant treatments was made through shared decision-making among the oncologist, the woman, and the accompanying persons. The survival endpoints which were evaluated included disease recurrence, contralateral breast cancers, new primary cancers, and death during the study period. The median follow-up was 101 months (ranging from 41 months to 190 months); 96% of the cohort had completed at least 5 years of follow-up by the time the study had ended, and 33% had completed 10 years of follow-up.

Statistical analysis. Frequencies and percentages were calculated to describe patient demographics. Correlation analyses to examine the association among disease recurrence, recommendation, and receipt of the systemic treatment were performed using the chi-squared test, *t*-test, and one-way ANOVA, as appropriate.

Logistic regression models were constructed to identify independent predictors, where a full model was first created by including all potentially important explanatory variables and then by removing the variable with the smallest contribution to the model at each step until a final backward stepwise model was obtained. Kaplan–Meier survival analyses were used to analyse disease-free and overall survival; *p* values were obtained using the log-rank test. Analyses were conducted using the Stata package release 11.0 (Stata Corporation, 4905 Lakeway Drive, College Station, Texas 77845, USA). A two-tailed *p* value test was used in all analyses and a *p* value of less than 0.05 was considered to be statistically significant.

## 3. Results

### 3.1. Demographics and Outcome of Women with T1N0M0 Cancers

We evaluated a total of 643 women diagnosed with node-negative stage I (T1N0M0) tumours over a 10-year period from 2006 to 2016. Details are shown in Table 1. The median patient age was 58 years (ranging from 28 years to 82 years) and 71% of the women (453 of 643) were post-menopausal. All women had undergone curative surgery, and none had received neoadjuvant treatment. A total of 339 (52.7%) women were treated with a mastectomy and 304 (47.3%) were treated with wide local excision (WLE); of these 304 women, 273 women (89.8%) completed whole breast radiation. Adjuvant chemotherapy was discussed with 334 women (51.9%) and about half of these women (169 of 334, 50.6%) opted to receive chemotherapy, with the majority (153 of 169, 90.5%) completing the recommended regimen; 71 of these women had HER2-positive tumours and also received trastuzumab. Ninety percent (457 of 506, 90.3%) of the women with a ER-positive disease received hormonal therapy.

The median follow-up was 101 months (ranging from 41 months to 190 months). Disease recurrence occurred in 30 of the 643 women (4.7%); 18 were isolated locoregional recurrences and 12 were systemic recurrences. Survival was excellent, with 5-year and 10-year recurrence-free survival reaching 96.6% and 95.5%, respectively. Seventy-three percent of the recurrences (22 of 30) had developed within 5 years, after a median interval of 29.5 months (ranging from 5 months to 59 months). Another eight women developed recurrence after 5 years: four were locoregional and four were systemic. There was a non-significant trend towards recurrences after 5 years being systemic in nature rather than locoregional (*p* = 0.384). Eighteen women had locoregional recurrences but no systemic disease, either at the time of re-staging when the recurrence was detected or later on throughout the duration of the follow-up. Twelve recurrences arose in the ipsilateral breast, four on the chest wall post-mastectomy, and two in the ipsilateral axilla. By stratifying these by tumour subtype, it was found that 10 women had ER-positive/HER2-negative diseases (three had received chemotherapy), three women had HER2-positive tumours (one received chemotherapy/trastuzumab), and five women had triple-negative tumours (four received chemotherapy). Two women experienced both locoregional and systemic recurrences: one woman was discovered with an ipsilateral breast recurrence after completing chemotherapy and 5 years of hormonal therapy and developed bone metastases 31 months later. Another woman presented with chest wall recurrences, and lung metastases were found on re-staging scans; she had previously declined all adjuvant treatments after the mastectomy. All 12 women with systemic recurrences, including the two women with concurrent locoregional recurrences, had ER-positive/HER2-negative tumours and three of them had received chemotherapy.

During the same period, 23 women (3.4%) developed contralateral breast cancer. Unlike recurrences, more contralateral cancers were detected after 5 years (nine contralateral cancers in the first 5 years compared to 14 contralateral cancers after 5 years). Six further women were diagnosed with a second new primary cancer that was unrelated to the breast cancer syndromes. Eight deaths (1.2%) were recorded by the end of the follow-up period, but only two were documented as being breast-cancer specific, both occurring in women with systemic recurrences.

### 3.2. Factors Associated with Disease Recurrence

We evaluated factors associated with disease recurrence. The mode of surgical treatment emerged as the most significant predictor of recurrence. Women treated with WLE alone had the highest likelihood of disease recurrence, as compared to women who had received both WLE and radiation (*p* = 0.022, OR 0.277, 95% CI 0.092–0.830) and those treated with a mastectomy (*p* = 0.001, OR 0.122, 95% CI 0.037–0.399) (Table 2). Women who had received whole breast radiation after WLE had recurrence rates that were comparable to those of women treated with a mastectomy (*p* = 0.112, OR 0.308, 95% CI 0.720–1.318). We looked more closely at the 304 women treated with WLE. Ninety percent (273 of 304) received post-WLE whole breast radiation with a tumour bed boost. A 10 Gy tumour bed boost was the standard, with 27 women receiving a higher tumour bed boost of 16 Gy: 22 women received it because of involved margins and five women received it because of close margins (tumours smaller than 1 mm away from the inked margin). One of these 27 women developed an ipsilateral breast cancer. She had a T1cN0 triple tumour, had declined repeat surgery for involved margins, but had completed chemotherapy and radiation (with a 16 Gy boost). There were 31 women who declined radiation after WLE, all of whom had adequate surgical margins; two women (6.4%) later developed ipsilateral breast recurrences. In comparison, 12 of the 273 women (4.4%) who received radiation therapy after WLE developed ipsilateral breast recurrences. Approximately half of the women (165 of 304) treated with WLE were offered chemotherapy; 78 women received chemotherapy (91.0% of these also completed radiation) and 87 declined the chemotherapy treatment (90.8% of these also completed radiation).

**Figure 1:**
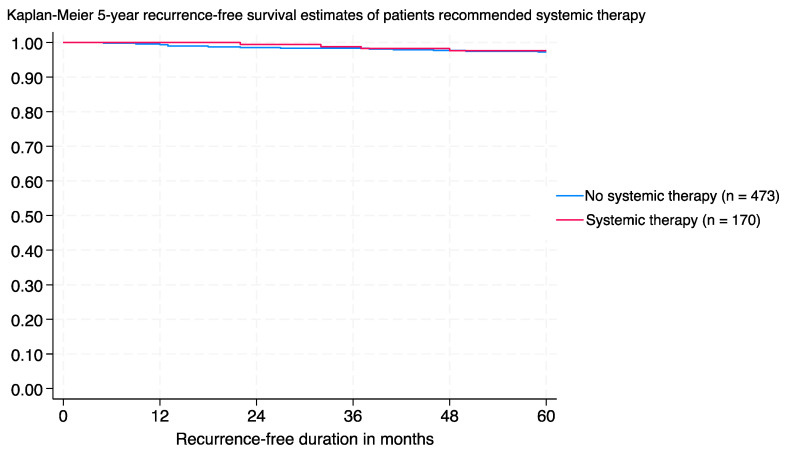
Kaplan–Meier survival curves of 5-year recurrence-free survival of T1N0 breast cancer patients stratified by systemic treatment (chemotherapy with or without targeted therapy) (*n* = 643, *p* = 0.444, HR 1.034, 95% CI 0.402–2.664).

Age at diagnosis, tumour subtype, and tumour size all showed no significant association with recurrence. Chemotherapy, with or without targeted therapy, and hormonal therapy both showed no significant association (*p* > 0.050) either (Table 1). The five-year recurrence-free survival was similar between those who were treated with chemotherapy (and/or targeted therapy) and those who remained untreated (97.6% versus 96.8%, respectively; *p* = 0.444, HR 1.034, 95% CI 0.402–2.664) (Figure 1). A similar trend was also observed in the subgroup of 334 women who were deemed to be potential candidates for chemotherapy by their oncologists (*p* = 0.767, HR 0.968, 95% CI 0.242–3.873) (figure not shown). No overall survival difference between treated and untreated women was observed (figure not shown).

### 3.3. Recommendation for Chemotherapy

At the multidisciplinary board meetings, 334 women (51.9%) were deemed to be at high risk of recurrence; therefore, chemotherapy, with or without anti-HER2 therapy, was recommended. These women tended to be young (*p* < 0.001) and pre-menopausal (*p* < 0.001, OR 1.799, 95% CI 1.273–2.540), and were more likely to have T1b or T1c tumours (*p* < 0.001, OR 0.025, 95% CI 0.011–0.059) (Table 3). The molecular subtype was another significant consideration, and women with HER2-positive or triple-negative tumours were four times more likely to be recommended chemotherapy compared to women with hormone-responsive HER2-negative tumours (*p* < 0.001, OR 0.268, 95% CI 0.184–0.391). Multigene genomic testing was conducted in 18 of the 459 women with HR-positive/HER2-negative tumours: Oncotype DX in 16 women, MammaPrint in one, and EndoPredict in another. Multigene testing returned a low score in 14 women (87.5%); two women had a high Oncotype recurrence score and two had a borderline Oncotype recurrence score. Chemotherapy was discussed with these four women and, eventually, one woman with a high recurrence score and one woman with a borderline recurrence score opted to receive chemotherapy.

### 3.4. Chemotherapy Treatment and Outcomes

Half of the women with whom chemotherapy had been discussed opted for chemotherapy (169 of 334, 50.6%); 42.0% (71 of 169) of these women had HER2-positive tumours and received trastuzumab concurrently. We observed that women who opted to receive chemotherapy, alone or in combination with anti-HER2 therapy, tended to be young (*p* < 0.001) and were more likely to have HER2-positive or triple-negative tumours (*p* < 0.001) (Table 4). Similar proportions of women with T1b and T1c tumours (*p* = 0.308) and similar proportions of women treated with WLE or a mastectomy opted for chemotherapy (*p* = 0.230). Overall, 14.6% (20 of 137) of all the women with T1b tumours and 39.3% (146 of 372) of all the women with T1c tumours received chemotherapy with or without anti-HER2 therapy. By subtype, 14.2% (65 of 459) of all the women with HR-positive/HER2-negative tumours received chemotherapy, compared to 43.4% (33 of 76) of all the women with triple-negative tumours and 65.7% (71 of 108) of all the women with HER2-positive tumours.

Next, we compared outcomes within this cohort of 334 women who were deemed to be chemotherapy candidates: 98.2% of the women in this cohort had T1b or T1c tumours, 26.6% had HER2-positive tumours, and 14.1% had triple-negative tumours. Similarly to what had been previously observed in the entire cohort, the clinical outcomes were similar between those treated with chemotherapy (and/or anti-HER2 therapy) and those who remained untreated (Table 4). Recurrences developed in 16 of these 334 women: four women with triple-negative tumours, all of whom developed locoregional recurrences after completing chemotherapy, one woman with HR-positive/HER2-positive diseases, who developed locoregional recurrences after chemotherapy/trastuzumab, and 11 women with ER-positive/HER2-negative tumours, six of whom had received chemotherapy and developed three locoregional and three systemic recurrences and five of whom had declined to undergo chemotherapy and developed two locoregional and three systemic recurrences. Four of the 11 women with ER-positive/HER2-negative tumours had completed 5 years of hormonal treatment and six others had relapsed while still on hormonal therapy; one woman had declined hormonal therapy as well as chemotherapy. Locoregional recurrences were observed to be more frequent among women who received chemotherapy (eight out of 169 women treated with chemotherapy versus two out of 165 who remained untreated, *p* = 0.104) and could not be attributed to more women in the chemotherapy group who had been treated with WLE. Similar numbers of women treated or untreated with chemotherapy developed systemic recurrences. Contralateral cancers were more common among women treated with chemotherapy, but this was not significant (*p* = 0.379). There were four deaths in this group and all occurred in women who had not received chemotherapy, with non-breast-cancer-related causes recorded in relation to three of them.

## 4. Discussion

Our study reaffirms the favourable prognosis observed in women with stage I tumours, where long-term disease-free survival is the norm rather than the exception [5,9]. In our cohort, we observed a 5-year recurrence-free survival of 96.6% and a 10-year recurrence-free survival of 95.5%. Recurrences occurred in 4.7% of the women in our study and, in two-thirds of these women, the disease was restricted to locoregional sites, such as the ipsilateral breast, chest wall, or axillary nodal basin, and was surgically resectable. Systemic diseases were uncommon even in the instance of locoregional recurrences; one woman with a chest wall recurrence was found, through re-staging scans, to have developed lung metastases, while the other woman developed bone metastases 31 months after developing an ipsilateral breast recurrence. It is, thus, unsurprising that local treatment emerged as the only significant predictor of disease recurrence through multivariate analysis. Women who did not receive whole breast radiation after WLE experienced the highest risk of recurrence, re-affirming the importance of whole breast radiation after WLE. Adequate surgical margins did not preclude the need for radiation, and 6.4% of the women who forwent radiation developed ipsilateral breast recurrences, despite all exhibiting adequate surgical margins and despite having been treated with chemotherapy and/or hormonal therapy. In comparison, recurrences occurred in 4.3% of the women who had completed a radiation treatment. Recurrence rates in women who had received radiation therapy after WLE were equivalent to those observed in women treated with a mastectomy, once again proving that outcomes after breast conservation and mastectomies are equivalent [10].

The majority of the recurrences occurred in the first 5 years following diagnosis, and we observed that recurrences after 5 years were more often systemic in nature, though no further conclusions could be drawn given the small number of recurrence events in our cohort. Existing data show that recurrence patterns vary among cancer subtypes: ER-positive tumours, both luminal A and B, show an increase in recurrence risk over time, while triple-negative and HER2-positive tumours tend to recur within the first 5 years [11,12,13]. We observed that recurrences were most frequent among women with triple-negative tumours (7.9%), followed by women with ER/PR-positive/HER2-negative tumours (4.5%), and were least frequent among women suffering from HER2-positive tumours (2.7%). It has been similarly reported that the recurrence risk is highest amongst women suffering from triple-negative tumours [14,15], although many have reported recurrences to be more frequent in HER2-positive tumours compared to ER-positive/HER2-negative tumours [16,17]. All 12 instances of systemic recurrences were found in women with ER-positive/HER2-negative tumours, a finding which is unusual, as distant metastases have been reported to be least common in luminal A tumours [17,18]. On the other hand, all recurrences in women with triple-negative tumours were locoregional, a finding which is also interesting given that triple-negative tumours have a propensity for early distant recurrence [13]. We are uncertain of the reason for the differences in the pattern of recurrences observed in our cohort, whether recurrence patterns in early node-negative T1 cancers differ from those observed in women suffering from more advanced diseases. Existing reports do not stratify recurrence patterns by disease stage, and most published studies do not look specifically at T1N0 tumours, unlike ours. The low incidence of systemic recurrences in our study is not unexpected, given that the investigated tumours were small node-negative cancers. The frequency of systemic recurrences observed in women suffering from ER-positive/HER2-negative diseases could be ascribed to the fact that only 14% of these women received chemotherapy, whereas 40% of the women with triple-negative tumours and 65% of the women with HER2-positive tumours received chemotherapy. Another plausible explanation could also be that these ER-positive/HER2-negative tumours were of the luminal B subtype or of a kind that would return high recurrence scores on genomic assays.

In our study, we found that chemotherapy was more often recommended to younger women, something which was expected since these women are generally expected to tolerate chemotherapy better and the chemo-benefit is perceived to be more significant given their longer life expectancy [19,20]. Similarly to other studies, chemotherapy was more often recommended to women with HER2-positive and triple-negative tumours because of the higher recurrence risk associated with such tumours, the proven effectiveness of anti-HER2 therapies, and the lack of any alternative systemic treatment options for triple-negative diseases [18,21]. On the other hand, many women with hormone-responsive diseases perceive hormonal therapy as an alternative to chemotherapy, and oncologists themselves also tend to push less strongly for chemotherapy since the chemo-response is less marked in ER-positive/HER2-negative disease [22]. Our study cohort was from a time period when genomic assays were not widely used, primarily because the assay was not covered under government subsidies or insurance plans. Only 18 women in our study, representing only 4% of the eligible women, had undergone genomic testing. The assay, Oncotype DX in most cases, returned a low score in 78% of the cases. Since then, insurance coverage has been extended to include genomic assays, and it has now become routine to offer genomic assay testing to eligible women. Even so, current uptake rates average at only about 49%, and many women still decline chemotherapy outright (unpublished data). There are suggestions of anti-HER2/chemotherapy benefits in women with node-negative T1a tumours from both prospective and retrospective studies, but the recruited numbers are small [3,23,24]. Some oncologists will discuss treatment with younger women deemed to present numerous risk factors, such as a high tumour grade, multifocality, and an ER/PR-negative status, but, generally, chemotherapy and anti-HER2 treatment would not be offered to women with T1aN0 HER2-positive disease. In our study, whether the woman had been treated with a mastectomy or WLE did not influence the consideration for chemotherapy, and almost equal numbers of women receiving chemotherapy had undergone WLE or a mastectomy. This contrasted with the common perception amongst many of the women in our cohort that a mastectomy was a ‘complete treatment’ and that adjuvant treatments were, thus, no longer necessary.

We next evaluated chemotherapy benefits and focused on the women who were thought to derive clinical benefits from chemotherapy. Almost all the women who were recommended a chemotherapy treatment had T1b or T1c tumours and, despite ours being a retrospective review, almost equal numbers of these women opted for or declined chemotherapy. Women in the treated and untreated groups were similar in terms of age, ethnicity, tumour size, type of surgery treatment, and differed only with respect to the tumour subtype. More women with HER2-positive and triple-negative tumours were treated with chemotherapy, possibly due to a strong push from the oncologists based on data which consistently show chemo-benefits even in patients with small node-negative, triple-negative, and HER2-positive tumours [7,25,26,27]. Low chemotherapy uptake rates amongst women with hormone-positive/HER2-negative has also been reported elsewhere [3]. There was a non-significant trend towards isolated locoregional recurrences among the women treated with systemic chemotherapy and/or targeted therapy, a finding which could not be attributed to higher breast conservation rates. The occurrence of systemic recurrences, however, was similar between the groups; the small numbers did not allow us to adjust further for tumour subtype. Interestingly, the disease recurrences that developed in the four women with triple-negative tumours and in the one woman with a HER2-positive tumour were all limited to locoregional sites, with no systemic diseases detected either concurrently or at a later time. We also did not observe any significant differences in recurrence and survival outcomes amongst the women who had received chemotherapy (with or without targeted therapy) and those who had not, regardless of whether the analysis included the entire cohort or was limited to the subgroup who had been deemed potential chemotherapy candidates (and who were recommended chemotherapy). This appears to be somewhat in contrast to a study published of a large cohort of 75,139 women with T1N0 tumours from the SEER database, where chemotherapy was observed to confer a survival advantage to women with T1b and T1c tumours [28]. In our study, roughly equal numbers of women with T1b or T1c tumours proceeded with or declined the recommended chemotherapy. Chemotherapy benefits were observed more consistently across all T1c tumours, while, in women suffering from T1b tumours, benefits were reportedly observed mostly in relation to tumours with unfavourable characteristics, such as those associated with a grade 3, HER2-positive, and triple-negative status [28]. Other studies have reported no survival benefit from adjuvant chemotherapy in patients suffering from T1mic, T1a, T1b triple-negative tumours [6,29]. An observational, non-randomised, prospective cohort study from within the NCCN database has reported excellent outcomes in relation to T1a and T1b tumours without chemotherapy treatment. Five-year disease-free survival was 94% to 96% for women suffering from T1b tumours treated with chemotherapy, which was no different from the Five-year disease-free survival of 90% to 96% for women suffering from T1b tumours who did not undergo chemotherapy [3]. We observed that deaths were more common in women who did not receive chemotherapy, though this was most likely because these women were older, more frail and had more co-morbidities, making them poor candidates for chemotherapy in the first place. The observation that most deaths were reported to be unrelated to breast cancer supports this. A recent report has also found non-cancer deaths to be more frequent among persons older than 65 years diagnosed with early-stage cancer [30].

## 5. Conclusions

Our study confirms the good outcomes observed in women with T1N0 tumours. Survival is excellent for these women, and disease recurrence is often limited to locoregional sites. Systemic recurrence was uncommon and occurred exclusively in women with ER-positive/HER2-negative tumours. The mode of local surgical treatment emerged as the only independent predictor of recurrence, and wide local excision, without whole breast radiation, was associated with the highest recurrence risk. Systemic chemotherapy (with or without targeted therapy) did not produce significant differences in recurrence events, contralateral cancers, or deaths.

## Figures and Tables

**Table 1:** Correlation analyses stratified by disease recurrence (*n* = 634). * HR+/HER2+ includes women with either ER- or PR-positive tumours, HER2+ includes all women with HER2-positive tumours regardless of ER/PR status, and ER-/PR-/HER2- includes all women with triple-negative tumours. ER: oestrogen receptor, PR: progesterone receptor.

	Women with Disease Recurrence (*n* = 30)	Women Remaining Disease-Free (*n* = 613)	*p* Value
Median age (years)	55 (40–77)	58 (28–86)	0.379
Menstrual status			0.382
Pre-menopausal	11 (36.7%)	179 (29.2%)	
Post-menopausal	19 (63.3%)	434 (70.8%)	
Ethnicity			0.839
Chinese	27 (90.0%)	538 (87.8%)	
Malay	2 (6.67%)	36 (6.20%)	
Indian	1 (3.33%)	23 (3.75%)	
Others	0 (0.00%)	16 (2.61%)	
Surgery			0.029
Wide local excision	20 (66.7%)	284 (46.3%)	
Mastectomy	10 (33.3%)	329 (53.7%)	
T status			0.649
T1mi	2 (6.7%)	25 (4.1%)	
T1a	6 (20.0%)	71 (11.6%)	
T1b	1 (3.3%)	93 (15.2%)	
T1c	21 (70.0%)	245 (40.0%)	
Median invasive size (mm)	13 (0–20)	12 (0–20)	0.032
Median tumour size (mm)	20 (3–60)	16 (1–120)	0.047
Subtypes *			0.437
HR+/PR+/HER2-	21 (70.0%)	438 (71.5%)	
HER2+	3 (10.0%)	105 (17.1%)	
ER-/PR-/HER2-	6 (20.0%)	70 (11.4%)	
Chemotherapy			0.380
Not received	20 (66.7%)	453 (73.9%)	
Received	10 (33.3%)	160 (26.1%)	
Hormonal therapy			0.217
Not received	11 (36.7%)	162 (26.4%)	
Received	19 (63.3%)	451 (73.6%)	

**Table 2:** Logistic regression stratified by disease recurrence in women with T1N0 tumours (*n* = 643). WLE: wide local excision, RT: post-operative radiation.

Variable	Level	Odds Ratio	*p* Value	95% CI
Median tumour size	-	1.019	0.063	0.999–1.040
Surgery	WLE alone WLE + RT Mastectomy	Reference 0.277 0.122	Reference 0.022 0.001	Reference 0.092–0.830 0.037–0.399

**Table 3:** Correlation analyses of women who were recommended chemotherapy (with or without targeted therapy) or with whom chemotherapy was discussed versus those who did not require chemotherapy (*n* = 643). * HR+/HER2+ includes women with either ER- or PR-positive tumours, HER2+ includes all women with HER2-positive tumours regardless of ER/PR status, ER-/PR-/HER2- includes all women with triple-negative tumours. ER: oestrogen receptor, PR: progesterone receptor.

	Women Recommended Chemotherapy +/− Targeted Therapy (*n* = 334)	Women Not Recommended Chemotherapy +/− Targeted Therapy (*n* = 309)	*p* Value
Median age (years)	57 (28–74)	60 (31–86)	<0.001
Menstrual status			<0.001
Pre-menopausal	118 (35.3%)	72 (23.3%)	
Post-menopausal	216 (64.7%)	237 (76.7%)	
Ethnicity			0.463
Chinese	294 (88.0%)	271 (87.7%)	
Malay	23 (6.9%)	15 (4.9%)	
Indian	10 (3.0%)	14 (4.5%)	
Others	7 (2.1%)	9 (2.9%)	
Surgery			0.262
Wide local excision	165 (49.4%)	139 (45.0%)	
Mastectomy	169 (50.6%)	170 (55.0%)	
T status			<0.001
T1mi	0 (0%)	36 (11.7%)	
T1a	5 (1.5%)	93 (30.1%)	
T1b	46 (13.5%)	91 (29.4%)	
T1c	283 (84.7%)	89 (28.8%)	
Median invasive size (mm)	15 (1–20)	7 (1–20)	<0.001
Subtypes *			<0.001
HR+/HER2-	198 (59.3%)	261 (84.5%)	
HER2+	89 (26.6%)	19 (6.1%)	
ER-/PR-/HER2-	47 (14.1%)	29 (9.4%)	

**Table 4:** Correlation analyses stratified by chemotherapy with or without targeted therapy treatment in the 334 women who had been recommended chemotherapy (*n* = 334). * HR+/HER2+ includes women with either ER- or PR-positive tumours, HER2+ includes all women with HER2-positive tumours regardless of ER/PR status, ER-/PR-/HER2- includes all women with triple-negative tumours. ER: oestrogen receptor, PR: progesterone receptor.

	Women Treated with Chemotherapy With/Without Targeted Therapy *(n* = 169)	Women with No Chemotherapy or Targeted Therapy (*n* = 165)	*p* Value
Median age (years)	55 (28–84)	60 (35–83)	<0.001
Menstrual status			0.095
Pre-menopausal	67 (39.6%)	51 (30.9%)	
Post-menopausal	102 (60.4%)	114 (69.1%)	
Ethnicity			0.651
Chinese	145 (85.7%)	149 (90.3%)	
Malay	14 (8.2%)	9 (5.5%)	
Indian	6 (3.6%)	4 (2.4%)	
Others	4 (2.4%)	3 (1.8%)	
Surgery			0.230
Wide local excision	78 (46.2%)	87 (52.7%)	
Mastectomy	91 (53.8%)	78 (47.3%)	
T status			0.736
T1mi	0 (0.0%)	0 (0.0%)	
T1a	3 (1.8%)	2 (1.2%)	
T1b	20 (11.8%)	26 (15.8%)	
T1c	146 (86.4%)	137 (83.0%)	
Subtypes *			<0.001
HR+/HER2-	65 (38.5%)	133 (80.6%)	
HER2+	71 (42.0%)	18 (10.9%)	
ER-/PR-/HER2-	33 (19.5%)	14 (8.5%)	
Disease recurrence			0.578
Isolated locoregional recurrence	8 (4.7%)	2 (1.2%)	
Systemic recurrence	3 (1.8%)	3 (1.8%)	
No recurrence	158 (93.5%)	160 (97.0%)	
Contralateral breast cancer			
Yes	7 (4.1%)	4 (2.4%)	
No	162 (95.6%)	161 (97.6%)	
Death			0.379
Breast-specific	0 (0.0%)	1 (0.6%)	
Other causes	0 (0.0%)	3 (1.8%)	

## Data Availability

Data is available on request through the corresponding author.
